# *Aedes aegypti* oviposition‐sites choice under semi‐field conditions

**DOI:** 10.1111/mve.12670

**Published:** 2023-06-02

**Authors:** Mariana R. David, Rafael Maciel‐de‐Freitas, Martha T. Petersen, Daniel Bray, Frances M. Hawkes, G. Mandela Fernández‐Grandon, Stephen Young, Gabriella Gibson, Richard J. Hopkins

**Affiliations:** ^1^ Laboratório de Mosquitos Transmissores de Hematozoários Instituto Oswaldo Cruz Rio de Janeiro Brazil; ^2^ Department of Arbovirology Bernhard‐Nocht Institute for Tropical Medicine Hamburg Germany; ^3^ Natural Resources Institute University of Greenwich, Central Avenue, Chatham Maritime Kent UK

**Keywords:** Aedes, behaviour, chemical ecology, chikungunya, container productivity, dengue, insect ecology, vectorial capacity, Zika

## Abstract

Vector control is still the recommended approach to avoid arbovirus outbreaks. Herein, we investigate oviposition preferences of *Aedes aegypti* (Diptera: Culicidae) females under a semi‐field structure Rio de Janeiro, Brazil. For that, in Experiment 1, we used two settings: ‘Single items’, which included as containers drain, beer bottle, bucket, car tyre, water tank, and a potted Peace Lily (*Spathiphyllum wallisii*) in a saucer with water, or ‘Multiple containers’, as an urban simulation, in which one drain, two additional beer bottles, and an extra plant pot saucer were added. Experiment 2 (sensory cues) used five variations of potted plant, each one varying in the range of sensory cues known to attract gravid females to oviposition containers. Our results indicate that gravid *Ae. aegypti* prefer to oviposit close to the ground and in open water containers with organic compounds from plant watering. Domestic large artificial containers containing tap water received significantly fewer eggs, except for the car tyre, which exhibited as many eggs as the potted plant. We also show that visual (potted plant shape) and olfactory clues (odour of the plant or from water containing organic matter) were equally attractive separately as were these stimuli together.

## Graphical abstract

- Gravid *Ae. aegypti* prefer to oviposit close to the ground and in open water containers with organic compounds from plant watering.
- Domestic large artificial containers containing tap water received significantly fewer eggs, except for the car tyre, which exhibited as many eggs as the potted plant.
- Visual (potted plant shape) and olfactory clues (odour of the plant or from water containing organic matter) were equally attractive separately as were these stimuli together.

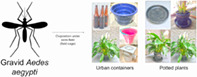

## INTRODUCTION

The mosquito *Aedes aegypti* is the primary vector of dengue (DENV), chikungunya (CHIKV) and Zika (ZIKV) viruses, particularly in urban settlements throughout tropical and subtropical areas. The number of dengue cases worldwide has increased over 8‐fold in the last two decades and approximately 390 million people are infected with DENV annually (Bhatt et al., 2013; Din et al., 2021). Furthermore, high levels of CHIKV and ZIKV infections have recently triggered unprecedented epidemics in Asia and America (Nunes et al., 2015; Zanluca et al., 2015). There are no vaccines available against CHIKV or ZIKV, so the focus of transmission reduction is vector control or interruption of human–vector contact.

Control of *Ae. aegypti* populations can be achieved through a combination of environmental management, chemical and biological control measures, and even more effectively through Integrated Vector Management (IVM) which involves all three approaches (Golding et al., 2015). Simple measures, such as (1) removing small containers (e.g., discarded cans and bottles which hold rainwater) and (2) covering large containers, such as water tanks, to prevent females from egg‐laying, especially in proximity to human dwellings, can reduce *Ae. aegypti* populations. These measures are encouraged, but little has been done to establish the optimal efficacy of breeding site elimination activities. Accurate information about *Ae. aegypti* breeding behaviour, including identification of the environmental and sensory cues associated with breeding sites can establish a basis for targeting the most productive breeding containers.

A combination of chemical and physical factors influences the oviposition behaviour of many mosquito species (Bentley & Day, 1989; Day, 2016). *Aedes aegypti* is well‐adapted to the urban environment and almost exclusively lays eggs in man‐made containers which hold relatively clean water, such as tyres, plant pots, plastic pots, drains and water tanks. Female *Ae. aegypti* generally deposit eggs on damp surfaces just above the waterline, such as the inner surfaces of discarded containers (Christophers, 1960; Clements, 1992; Cunha et al., 2002; David et al., 2021; de Abreu et al., 2015; Longdon et al., 2015; Maciel‐de‐Freitas, Marques, Peres, Cunha, & de Oliveira, 2007). In addition, this species engages in a behaviour known as ‘skip oviposition’, which means that a single female can distribute eggs from a single batch across several oviposition containers (Colton et al., 2003; de Abreu et al., 2015; de Jesus et al., 2020). These characteristics are likely to enhance the survival rate of the immature stages in this species by reducing intraspecific competition and avoiding exposing all offspring to abiotic stresses, breeding site drying and exposure to insecticides (Reiter, 2007).

In the field, a wide variety of artificial containers are colonised, but some are reported to produce a greater number of *Ae. aegypti* larvae than others (Barnes et al., 1995; Focks & Chadee, 1997). Previous studies have investigated factors that modulate *Ae. aegypti* oviposition in laboratory, semi‐field and field conditions (Chadee et al., 1990; de Abreu et al., 2015; Harrington et al., 2008; Kaw et al., 2004; Wong et al., 2011). Physical characteristics, such as size, shape and colour of a container, and environmental factors, such as water temperature, water turbidity and sunlight exposure have been shown to influence oviposition. Chemical factors, including concentration of organic matter and plant derived‐chemicals, salinity and the presence of conspecific larvae and pupae are also known to affect a female's choice of oviposition container (Barrera et al., 2006; Chadee et al., 1990; Harrington et al., 2008; Kaw et al., 2004; Ponnusamy et al., 2008; Wong et al., 2011; Wong et al., 2012). However, water itself may be the strongest stimulant of all for oviposition. It has been shown that *Ae. aegypti* can detect changes in relative humidity through receptors contained within antennal basiconic sensilla (Kellogg, 1970). Gravid mosquitoes may be preferentially attracted to containers with a large water surface area and unconstrained access to the water. Increasing container height above ground level may reduce oviposition: mosquitoes generally fly close to the ground where the strength of air currents is generally lowest, saving energy, avoiding being blown off‐course (Clements, 2011), and reducing predation by high‐flying predators.

The aims of the present study were (1) to identify the behavioural responses of gravid females to a range of typical domestic containers commonly found in Rio de Janeiro, Brazil under semi‐natural controlled choice‐test conditions (David et al., 2009; Focks & Chadee, 1997; Maciel‐de‐Freitas, Marques, Peres, Cunha, & de Oliveira, 2007), comparing the responses of females to a low vs. high density distribution of containers and (2) to assess the strength of response in gravid females to sensory cues associated with typical potted garden plants. By promoting a deeper understand of the oviposition site preferences of gravid *Ae. aegypti* we support the development of vector control guidelines targeting those containers more attractive to mosquito females.

## MATERIALS AND METHODS

### Mosquito rearing

*Aedes aegypti* mosquitoes (F2 generation) were derived from a laboratory colony established from eggs collected with ovitraps (Fay & Eliason, 1966) in Urca, Rio de Janeiro, Brazil (22°56′43″ S; 43°09′42″ W). Larvae were fed with commercial fish food (Tetramin®). Males and females were maintained in the same cage for 72 h after emergence under laboratory conditions (25 ± 2°C and 70 ± 10% RH) to allow mating to occur, and they were fed ad libitum with 10% sucrose. Three‐day old females were blood‐fed using the Hemotek® membrane feeding system (Discovery Workshops, Accrington, UK) with human blood. The use of human blood was approved by the Fiocruz Ethical Committee (process CAAE 53419815.9.0000.5248).

### Semi‐field cages

Oviposition assays were conducted in outdoor field cages at Fundação Oswaldo Cruz campus, Rio de Janeiro, Brazil (de Jesus et al., 2020). Cages consisted of an outdoor rendered space (16 m × 11 m × 2.5 m high) surrounded on three sides by nylon netting and the fourth side consisted of a brick wall and entrance doors. The ceiling/roof was also made of brick. The space was sub‐divided with nylon netting partitions into six smaller field cages (5 m × 4 m × 2.5 m high), though only three of these field cages (cages 02, 03 and 06 from de Jesus et al. (2020) were used at one time in this study. Experiments were conducted during the spring season (October–November) 2016. Temperature and relative humidity (RH) records were obtained from the nearest meteorological station, ~13 km from the field cages, and ranged between 19°C and 32°C, and 49%–92% RH. Fifty mated female *Ae. aegypti* (6–7 days‐old) were released in the centre of the cage two meters above the ground into each field cage 3 days after receiving a bloodmeal (the time needed for egg maturation).

**FIGURE 1:**
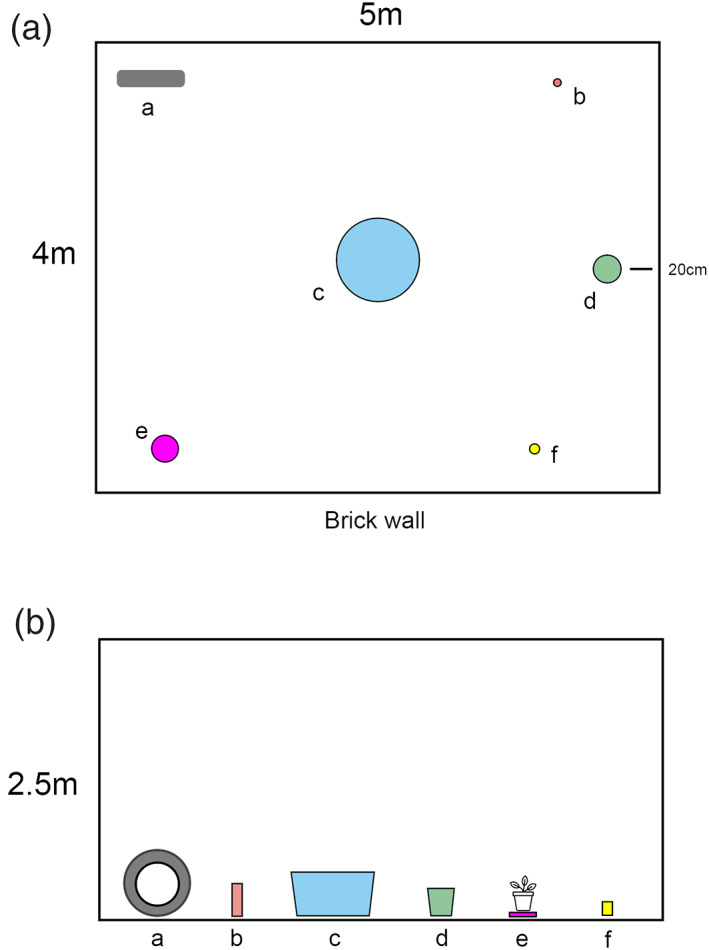
Schematic diagrams of each domestic container in Table 1 to scale in semi‐natural experimental field cage showing ‘Bird's eye view’ (A) and side view (B) of positions of six types of container, showing relative size and shape of containers within semi‐field cages. All containers were filled with tap water. Top view indicates relative distances between containers and cage walls, and the size of each container's opening. Bottom view indicates relative heights of each container's opening and relative distances to the top of the cage, but horizontal spacing is not accurate. a: Tyre; b: Bottle; c: Water Tank; d: Bucket; e: Plant pot saucer; f: Drain. Table 1 shows the actual containers used and their dimensions.

The main experimental protocol consisted of distributing a range of potential oviposition containers filled with water throughout each field cage, as shown in Figure 1 and Table 1 for Experiment 1.

**TABLE 1:** Container dimensions, and relative accessibility of domestic containers to gravid females.

Container			Width of orifice (cm)	Height of orifice above floor (cm)	Exposed surface area of water (cm^2^)
Chart legend (Figure 2)	Description				
Drain	Drain (side port blocked, access only via top grille)	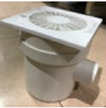	<1	10	95
Bottle	Beer/soft drink Bottle	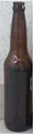	3	28.6	31.5
Water tank	Water tank	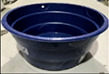	73	41	4183
Bucket	Bucket	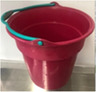	25	26	491
Tyre	Tyre	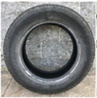	60	15	589
Saucer	Plant pot saucer	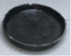	25	3	625
Plant saucer	Plant pot saucer under Peace Lily plant	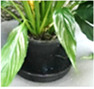	25	3	290

The mosquitoes were given 1 week to oviposit in the field cage, after which all oviposition containers were moved to an insectary with controlled temperature (25 ± 2°C). Water levels in containers were increased to submerge eggs oviposited above the water surface and 500 mg of dry yeast was added to the water to ensure larvae had adequate food to develop normally. Eggs are too small and too well‐camouflaged against the container surfaces to be counted reliably, so we waited until larvae matured to the L3–L4 stages, and then counted these larvae daily for a week. Although we did not directly count the eggs, we believe that the ideal rearing conditions led to minimal larval mortality and these data can be used as a proxy for the number of eggs laid initially. As soon as a larva was counted, it was removed from the container. Container inner surfaces were sponge washed before use in the next experiment to remove any remaining eggs.

### Experimental design

*Aedes aegypti* oviposition choice across a wide range of typical breeding sites including a variety of domestic containers and garden potted plants was compared in different settings to identity factors that favour higher oviposition rates of *Ae. aegypti* mosquitoes.

*Experiment 1a (Single items)*: investigated oviposition choice across typical domestic containers; drain, upright beer/soft‐drink bottle, bucket, upright car tyre and a water tank, against a control of potted plant (Peace Lily *Spathiphyllum wallisii*). The choice of this plant for the experiments was due to its occurrence in domestic gardens in the study area and adaptation to shaded areas such as the field cages. The ‘single items’ setting included one each of the containers shown in Table 1 positioned as indicated in Figure 1.

*Experiment 1b (Multiple containers)*: The same range of containers and the same protocols were followed, except an additional drain, two additional beer/soft‐drink bottles, and an additional plant pot saucer were included with the original set of containers in the semi‐field cages to simulate the greater density of small containers in urban areas. Equal containers were positioned next to each other in the positions indicated in Figure 1. The number of gravid females was the same in both experiments (*n* = 50). The aim was to determine whether density of containers affects egg‐laying behaviour.

**FIGURE 2:**
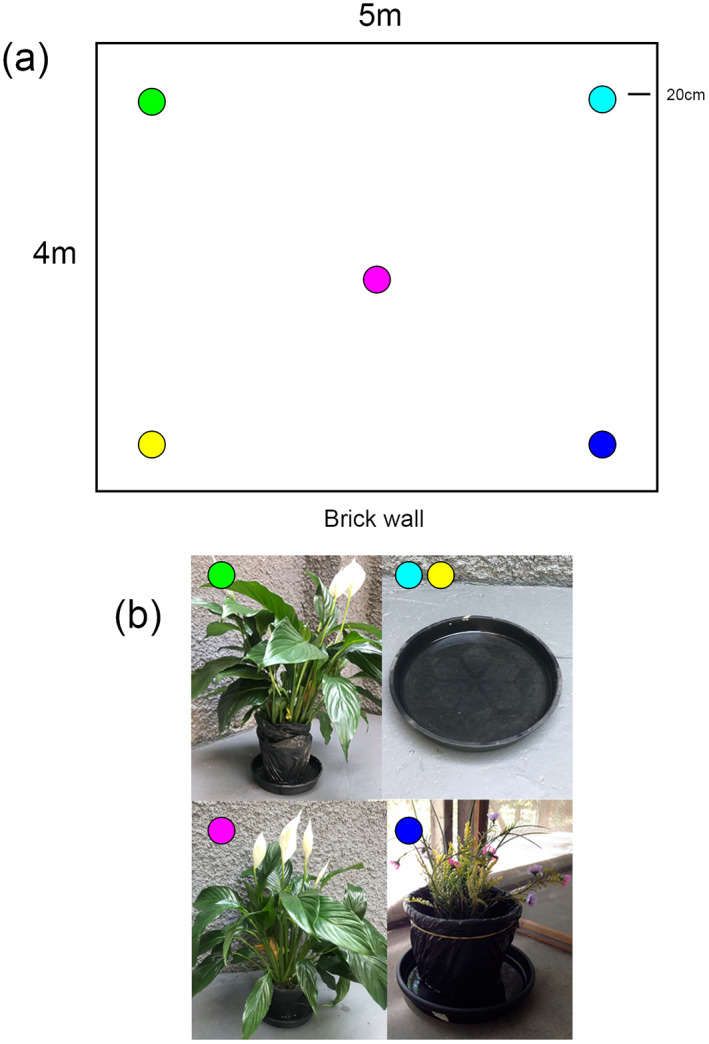
Schematic diagrams of each domestic container in Table 2 in semi‐natural experimental field cage showing ‘Bird's eye view’ of positions five types of containers (A) and actual containers used (B). Green circle: ‘Bagged plant’; Cyan circles: ‘Plant water’; Pink circle: ‘Plant’; Yellow circle: ‘Tap water’; Dark blue circle: ‘Artificial Plant’. Table 2 describes container characteristics.

*Experiment 2 (Sensory cues)*: investigated the responsiveness of gravid females to the following key sensory cues associated with oviposition in common garden potted plants; visual (appearance of the plant and container), olfactory (odours associated with the plant, the soil and the water) and water quality (salinity, pH and other factors). In each case, water was in the plant pot saucer, and five variations were tested, providing a range of sensory cues known to attract gravid females to oviposition containers (Table 2). Saucers were positioned in the field cages as indicated in Figure 2.

**TABLE 2:** Experiment 2 treatments.

Treatment	Description
Tapwater	Tap water in saucer
Bagged plant	Plant in a bag & tap water in saucer (visual and olfactory cue)
Artificial plant	Plastic plant & tap water in saucer (visual cue)
Plant water	Saucer with water drained from plant (olfactory cue)
Plant	Plant in a saucer with water drained from plant (visual and olfactory cues)

*Note*: In each case, the container was a plant pot saucer, and the plant was a Peace Lily. The treatments “Bagged plant” and “Plant” both also have the olfactory clues from the vegetation of the plant beyond those associated with the egg laying sites (i.e., from the water).

Each treatment was designed to test the responsiveness to the following sensory cues; vision (e.g., size, shape, contrast) of potential oviposition site, olfactory (volatile odours associated with oviposition site) and water vapour alone (i.e., untreated tap water vs. water plus volatile organic compounds).

A ‘Plant’ was composed by a potted Peace Lily as used in Experiment 1. The water in the saucer came from plant irrigation with tap water.

An ‘Artificial plant’ treatment was composed of an artificial plastic plant in a pot without soil, placed in a saucer of tap water. This treatment provides an approximation of the visual cues associated with a live plant but lacking in chemical cues.

The ‘Bagged plant’ treatment consisted of a live plant in a pot of gardener's soil contained in a plastic bag which prevented the soil and water in the plant pot from contacting the saucer of tap water under the pot, which is where gravid females normally oviposit. This treatment was designed to determine the strength of response to the general plant odours emanating from the bagged plant, given that tap water in the saucer would lack organic olfactory components.

The ‘Tap water’ treatment, consisting of a plant pot saucer containing tap water only, was included to determine the strength of attraction to this single oviposition attractant, that is, lacking in plant‐associated visual or chemical cues.

The ‘Plant water’ treatment was the same as Tap water, except the saucer contained water which had been drained from the pot of a Peace Lily, providing chemical cues associated with a live plant (soil, water and organic material in the soil), but no visual cues associated with plants.

These treatments were chosen to determine whether these sensory cues had a significant effect on the oviposition behaviour of gravid females. As for Experiment 1, one of each of the five types of experimental containers was placed in each of three field cages and the treatments were evenly distributed in the field cages and 50 blood‐fed female mosquitoes were released into the cage and allowed 1 week to lay eggs. The number of eggs laid in each container type was assessed by larval counts, as for Experiment 1.

### Statistical methods

The studies all involved side‐by‐side comparison of different containers in the same cage, with the same group of 50 insects. Experiment 1 had replicate data from 18 cages, and Experiment 2 from 6 cages. We investigated the potential for intra‐cluster correlation within cages using the Experiment 1 data. The intra‐cluster correlation coefficient was very small: 0.0056—resulting in a trivial design effect. Thus, larval counts from individual containers can serve as true replicates.

All subsequent analyses used generalised linear models (GLMs) with a log link and quasi‐poisson error distribution. GLMs with negative binomial errors did not converge, and poisson GLMs were grossly over‐distributed.

Stable multiple comparisons were obtained from linear models using log‐transformed counts, with a holm‐corrected LSD test (R, agricolae package). Experiment 1 was a two‐way analysis of deviance and Experiment 2 was a one‐way analysis of deviance. Analysis of deviance tables and QQ plots for model residuals are in a statistical appendix.

## RESULTS

### Experiment 1 comparison of oviposition choice between types of container and between ‘single items’ vs. ‘multiple containers’

The average total number of larvae (counted for all the containers in a given cage) was 61% higher for the ‘Single items’ treatment cages: 1422 ± 181, versus 884 ± 99 for the ‘Multiple containers’ treatment cages (means ± SE). The data were analysed with average values for the cases where there were groups of the same container in the multiple‐container treatment, producing a single replicate for each container for each run, both for the single and the multiple treatments. Table 3 shows mean larvae numbers, with standard errors calculated from raw data, for each container type. There were statistically significant differences in larval numbers across container types (*F*_6,128_ = 57.92, *p* < 0.0001, for container main effect: Analysis of deviance, quasipoisson GLM). Table 4 has the multiple comparison groups from this study.

**TABLE 3:** Mean larvae numbers (±SE from raw data) for each container type, data from single item and multiple container treatments.

Container	Number of larvae (mean ± SE)	
	Single item	Multiple items
Drain	0.33 ± 0.33	1.83 ± 1.08
Bottle	43.67 ± 24.87	0.15 ± 0.11
Water tank	21 ± 13.68	20.22 ± 7.21
Saucer	‐	51.3 ± 17.9
Bucket	66 ± 22.61	81.67 ± 24.57
Tyre	538.78 ± 132.31	238.11 ± 35.66
Plant	759.22 ± 150.46	488.78 ± 90.46

**TABLE 4:** Multiple comparison table, from holm‐corrected LSD test based on linear model.

Treatment and container	Log‐scale mean	Groups
SingleItem‐plant	6.41	a
MultipleItem‐plant	6.05	a
SingleItem‐tyre	6.05	a
MultipleItem‐tyre	5.38	ab
SingleItem‐bucket	3.73	bc
MultipleItem‐bucket	3.41	c
MultipleItem‐saucer	3.01	c
MultipleItem‐watertank	2.50	cd
SingleItem‐bottle	2.16	cd
SingleItem‐watertank	1.59	cde
MultipleItem‐drain	0.60	de
SingleItem‐drain	0.15	e
MultipleItem‐bottle	0.11	e

The statistical model has main effects for two factors (**Container**, with 7 levels for each container tested, and **Treatment**, with two levels: *Single items* and *Multiple containers*), and an interaction term, **Container:Treatment**.

The box and whisker plot of larval numbers (Figure 2) shows three statistically distinct categories:

1. Low (average larvae number 1.1): Drain had consistently fewest larvae, even with the extra Drain added to the ‘Multiple containers’ treatment.
2. Medium (average larvae number 41.6): Water tank, Bottle, Tap water saucer, and Bucket had a mean of ~5 to 50 larvae per container, with significant differences between ‘Single items’ and ‘Multiple containers’ only for Bottle.
3. High (average larvae number 506.2): Plant Saucer and Tyre had more larvae than the rest of the containers. The Plant Saucer water surface area was only 290 cm^2^, compared to 589 cm^2^ for the Tyre (Table 1), which means the Plant saucer treatment exhibited twice as many larvae as the Tyre per unit water surface area.

A holm‐corrected multiple comparison test based on a linear model showed significant between these three groups: Low versus Medium, difference = 40.5, *p* < 0.001; Medium versus High, difference = 464.6, *p* < 0.001, and Low versus High, difference = 505.1, *p* < 0.0001.

### Experiment 2. Effect of visual and chemical cues on oviposition choice associated with various plant pot treatments

**FIGURE 3:**
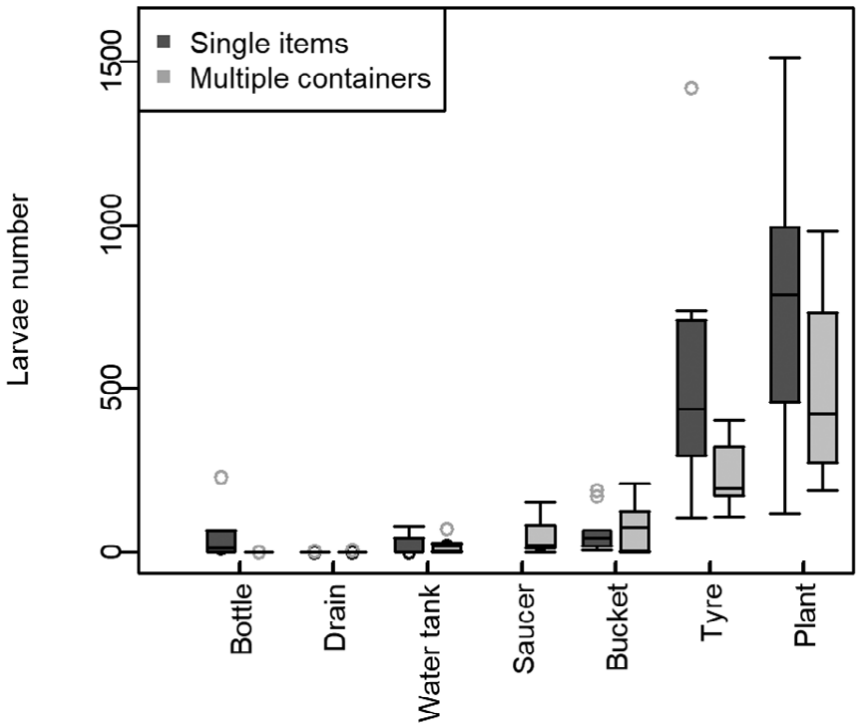
Experiment 1: ‘Single items’ and ‘Multiple containers’ treatments. Box and whisker plots showing larvae numbers for container types described in Table 1. The ‘Saucer’ container with no plant was only used in the urban simulation treatment. Except for Plant, all containers have tap water.

Figure 3 shows larval numbers in each of the plant pot saucer treatments described in Table 2 above, while Table 5 shows means ± SE (calculated from raw data) for each plant saucer treatment. Differences between treatments are statistically significant (*F*_4,25_ = 11.64, *p* = 0.00018, one‐way analysis of deviance, quasipoisson GLM).

**TABLE 5:** Means ± SEs for each plant saucer treatment.

Treatment	Number of larvae (mean ± SE)
Tap water	37 ± 20
Artificial plant	231.1 ± 61.6
Bag plant	219 ± 33.6
Plant water	334.7 ± 75.9
Plant	597.2 ± 106.8

The statistical model has a single factor, **Container**, with five levels, one for each container used.

A multiple comparison test showed that the saucer containing only tap water, with no plant (neither visual nor olfactory cues), showed a significant reduction in larval numbers when compared to any of the other treatments, all of which provided a visual or olfactory cue (or both). Table 6 has the results of each of the multiple comparisons.

**TABLE 6:** Multiple comparisons.

Comparison	Log‐scale difference	SE	*z* value	*p* value
Bag plant vs. artificial plant	−0.05	0.35	−0.15	0.99
Plant vs. artificial plant	0.95	0.29	3.31	0.007
Plant water vs. artificial plant	0.37	0.32	1.17	0.75
Tap water vs. artificial plant	−1.83	0.65	−2.79	0.04
Plant vs. bagged plant	1.00	0.29	3.43	0.005
Plant water vs. bagged plant	0.42	0.32	1.33	0.66
Tap water vs. bagged plant	−1.78	0.66	−2.70	0.048
Plant water vs. plant	−0.58	0.25	−2.29	0.13
Tap water vs. plant	−2.78	0.63	−4.43	<0.001
Tap water vs. plant water	−2.20	0.64	−3.43	0.004

**FIGURE 4:**
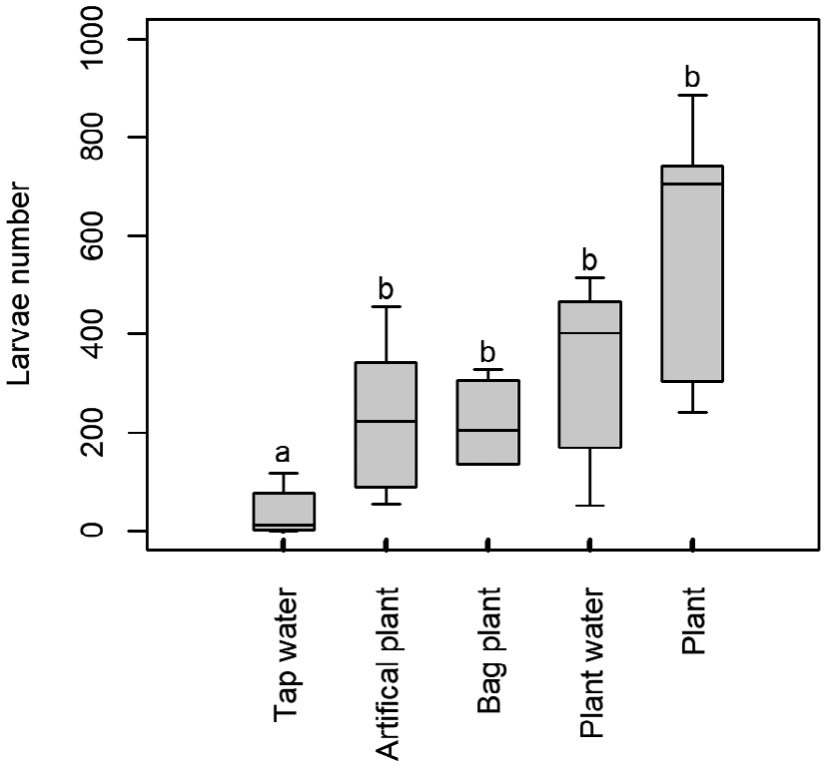
Experiment 2. Box and whisker plot of average larvae number per plant pot saucer container (treatments described in Table 2). Bars show standard errors from ANOVA model, and CLD letter codes from a multiple comparison test indicate groups of means which are not significantly different.

Either cue alone is enough to result in increased larval numbers. The ‘Plant’ treatment, with both visual and olfactory cues, had the largest average larval numbers, but was not significantly different from the single cue treatments (Figure 4).

## DISCUSSION

This semi‐field cage study of the oviposition site preferences of gravid *Ae. aegypti* demonstrates that they seem to prefer to lay eggs in containers with organic matter rather than in clean water and/or containers with water surface close to the ground and with easy access, for example tyres and plant pot saucers in comparison to drains with narrow openings in Experiment 1. In addition, when the attractiveness of key sensory stimuli associated with oviposition in common garden potted plants were tested (Experiment 2), gravid females were equally attracted to lay eggs in plant pot saucers by both visual and olfactory cues.

While the densities of potential egg laying containers vary greatly between sites in the field (Barnes et al., 1995; David et al., 2009; Focks & Chadee, 1997; Maciel‐De‐Freitas, Codeço, & Lourenço‐De‐Oliveira, 2007) and the removal of rubbish is a clear target for reducing vector reproduction, the role of plant pot saucers has been emphasised by the results of this study, highlighting the strength of attraction to water that has passed through organic matter in the soil. Other field‐based studies of gravid *Ae. aegypti* behaviour have shown that gravid females prefer to lay eggs in unmanaged containers that hold organic detritus and decaying leaves, which probably serve as a source of chemical attractants, indicating the presence of food for larvae (Barrera et al., 2006; Kaw et al., 2004; Wong et al., 2011; Wong et al., 2012).

It is possible that the attraction to water that came in contact with the *S. wallisii* plant substrate is related to plant/soil chemical agents, microorganisms or generic organic matter odours. Water vapour is known to influence oviposition container choice in mosquitoes, including *Ae. aegypti* (Bentley & Day, 1989; de Abreu et al., 2015). Chemical compounds associated with grass infusions, decaying leaves, conspecific larvae, predators and bacteria mediate oviposition behaviour, serving as attractants or repellents (Chadee et al., 1990; Ponnusamy et al., 2008; Torres‐Estrada et al., 2001; Trexler et al., 1998). These compounds may be detected by gravid females through either olfaction at a distance or through gustation on contact with the water. *Aedes aegypti* also prefer to lay eggs in containers with high turbidity and presence of organic detritus which may be due to visual and olfactory cues to locate breeding containers with sufficient food for their progeny (Kaw et al., 2004; Wong et al., 2011). Larval development rate and survival, in addition to emerging adult body size, have been positively associated with the amount of organic matter in natural breeding containers (David et al., 2021; Wong et al., 2012).

The choice of *S. wallisii* for the experiments was due to its occurrence in the study area and adaptation to shaded areas such as the field cages. Leaf infusions produced from different plant species differ in their potential to attract gravid mosquitoes (Ponnusamy et al., 2010; Santana et al., 2006) and their influence on oviposition behaviour has been linked to variation in metabolites produced by bacteria and other microorganisms (Ponnusamy et al., 2008). Further studies are necessary to access whether other plants would produce the same effects on the oviposition behaviour of *Ae. aegypti*. Furthermore, in the specific case of the plant saucers, plant‐watering can also cause nutrification of the water accumulated under the pot by leached soil fertilisers. Nitrogen‐phosphorous‐potassium (NPK) enrichment in oviposition container water has been shown to be significantly more attractive than distilled water to gravid *Ae. aegypti* and enhances the rate of larval development compared to plant material alone (Darriet, 2018). It was not possible to determine the fertiliser content of the plant substrate used in this study.

Our results have also shown that other containers close to the ground, with a relatively larger surface area, compared to that of a potted plant saucer, can be equally attractive to gravid females as the second most numerous larvae were found in the tyre, even though it contained clean water, confirming suspicions that urban debris is an important source of breeding sites for *Ae. aegypti* (Chan et al., 1971; Ferdousi et al., 2015). This attraction is probably related to the proximity to the ground and the size of the surface area of the water, which is large enough to provide a strong stimulus of water vapour, and easy access to the surface water and egg‐laying surfaces. Studies in urban environments generally conclude that *Ae. aegypti* lay eggs almost exclusively in artificial containers (Braks et al., 2003; Chan et al., 1971; Cunha et al., 2002; David et al., 2009; Maciel‐de‐Freitas, Marques, Peres, Cunha, & de Oliveira, 2007). Previous investigations in urban areas showed that this species has a high productivity in discarded tyres (Abílio et al., 2018; Stein et al., 2002), which also serve as a vehicle for the passive dispersion of eggs of *Ae. aegypti* and *Aedes albopictus* mosquitoes (Bennett et al., 2019; Hawley et al., 1987).

On the other hand, our results indicate that the largest containers received significantly fewer eggs, contrasting previous field data showing that water reservoirs for domestic use such as water tanks and buckets are the highly productive containers for *Ae. aegypti* (Barnes et al., 1995; Barrera et al., 1993; Maciel‐de‐Freitas, Marques, Peres, Cunha, & de Oliveira, 2007). Under natural conditions, those breeding sites are permanent and rarely washed, which frequently leads to some accumulation of organic matter and planktonic microorganisms' growth, factors positively associated to *Ae. aegypti* larvae abundance in this habitats (Garcia‐Sánchez et al., 2017; Overgaard et al., 2017). This characteristic could not be simulated in our study, since the water reservoirs were washed at each replicate of the experiments. This may have led to a lower oviposition rate in the water tank and bucket.

Curiously, the average total number of larvae (counted for all the containers in a given cage) was higher for the ‘Single items’ treatment cages than for the ‘Multiple containers’ treatment cages. The reduction of egg numbers in the more complex urban simulation environment was unexpected and may have been influenced by the climate. Although we alternated treatments between the weeks of experiments, we cannot exclude the possibility that fluctuations in temperature and humidity in the semi‐field could have influenced mosquito survival, behaviour and/or fecundity. Another possible explanation is that it may be a consequence of more confusing visual stimuli in the ‘Multiple containers’ treatment cages. Following the same logic, the ‘Multiple containers’ treatment used a group of three bottles, in which there were virtually no larvae, possibly because the group of bottles produced a confusing visual cue. Female mosquitoes rely on their vision to orient and find oviposition sites, using light reflection from the water surface and visual clues contrasting to the background to find potential breeding sites (Day, 2016; Kennedy, 1942).

In order to elucidate which characteristics of the potted plant saucer enhanced the attraction of gravid female *Ae. aegypti,* we tested the effects of separating visual and olfactory stimuli between this breeding container in Experiment 2. The ‘Plant water’ treatment (a saucer holding water that passed through plant subtract) was expected to hold more larvae than the other treatments in Experiment 2, but this difference was not statistically significant. Although the greater water surface area provided by the absence of a plant pot might have allowed a greater release of volatile compounds, this was not enough to enhance egg oviposition by gravid mosquitoes in this container. In the same way, gravid *Ae. aegypti* laid as many eggs in a saucer with an artificial potted plant as in a saucer holding a potted *S. wallisii*. These observations indicate that visual (potted plant shape) and olfactory stimuli (odour of the plant or the water containing organic matter) were equally attractive separately as these stimuli together. Thus, any of these attractants or a combination of them, along with the proximity to the ground, may have resulted in the greater attraction of gravid *Ae. aegypti* to the potted plant saucers compared to other artificial breeding sites in Experiment 1.

This conclusion contrasts the observation that chemical and physical cues act synergistically in influencing the ovipositional site selection by gravid *Aedes triseriatus* and *Culex* mosquitoes (Beehler et al., 1993; Dhileepan, 1997; McDaniel et al., 1976; Wilton, 1968). This discrepancy can be explained both by ecological differences between these mosquito genera, since *Ae. triseriatus* and *Culex* usually oviposit in natural containers (e.g., tree holes) (Aziz & Hayes, 1987) and water collections rich in organic matter (e.g., ponds, sinkholes and sewer ditches) (Liu et al., 2019), respectively; and by methodological differences (chemical attractants and breeding sites used in the experiments).

A limitation of the present study that must be considered is that containers were not rotated within the semi‐field cage in both Experiments. Although (Roque & Eiras, 2008) showed that the position of oviposition traps did not affect the capture rate of gravid *Ae. aegypti* in six out of eight cages in a similar field‐cage setting, we cannot eliminate the possibility that the ‘fixed position’ of containers might have biased the results. Microhabitat characteristics such as humidity, temperature, wind and proximity to resting places might influence mosquito oviposition (Sofia et al., 2019).

Investigating oviposition preferences of mosquitoes can lead to more effective measures for reducing *Ae. aegypti* productivity and more efficient strategies to reduce vector populations. Although our study is a simplification of the real world, for example, containers with tap water without debris, our results indicate that the removal of tyres and plant saucers from garden potted plants might be as effective as removing all domestic containers (with much less effort) to reduce arbovirus transmission. Since it is currently not possible to eliminate invasive mosquito species such as *Ae. aegypti* from large urban areas, outbreaks must be avoided by keeping the vector density below a critical transmission threshold (Focks, 2003). Moreover, physical control is still one of the most reliable tools to reduce vector populations (Morrison et al., 2008) but it is impossible to inspect and eliminate or manage all potential breeding containers. Thus, control and community engagement efforts must be directed to the most attractive water reservoirs to gravid *Ae. aegypti* (Barnes et al., 1995). In Rio de Janeiro, for example, during a larval survey conducted during the wet season, 28 tyres were found with water (only 0.8% of containers), which hold 455 larvae (16.3 larvae/breeding site). On the other hand, 1202 drains (33.1% of containers) were checked, in which 162 larvae of *Ae. aegypti* were collected (0.3 larvae/breeding site) (Maciel‐de‐Freitas, Marques, Peres, Cunha, & de Oliveira, 2007). Identifying the stimuli involved in oviposition container choice can help to design more effective traps for mosquito control and/or surveillance, such as insecticide dissemination stations (Devine et al., 2009).

## AUTHOR CONTRIBUTIONS

**Mariana Rocha David:** Conceptualization; investigation; methodology; visualization; writing – original draft; writing – review and editing. **Rafael Maciel‐de‐Freitas:** Conceptualization; funding acquisition; investigation; methodology; resources; writing – review and editing. **Martha Thieme Petersen:** Investigation; methodology; writing – review and editing. **Daniel Bray:** Formal analysis; writing – review and editing. **Frances Hawkes:** Investigation; writing – review and editing. **G. Mandela Fernández‐Grandon:** Investigation; writing – review and editing. **Stephen Young:** Formal analysis; writing – original draft; writing – review and editing. **Gabriella Gibson:** Conceptualization; formal analysis; visualization; writing – original draft; writing – review and editing. **Richard Hopkins:** Conceptualization; funding acquisition; methodology; writing – review and editing.

## CONFLICT OF INTEREST STATEMENT

The authors declare no conflicts of interest.

## Supporting information


**Data S1.** Supporting Information

**Data S2.** Supporting Information

## Data Availability

The data that support the findings of this study are available from the corresponding author upon reasonable request.
