# Supplementary material for: Aedes aegypti oviposition‐sites choice under semi‐field conditions
Source: Med Vet Entomol. 2023 Jun 2;37(4):683–92. doi: 10.1111/mve.12670 (PMC10946600; doi:10.1111/mve.12670)

**Statistical appendix**

|  |
| --- |

(Quasi distribution GLMs use F tests,

with the dispersion parameter as an estimate of the residual mean deviance)

**Experiment 1 – 2-way analysis of deviance, quasi-poisson GLM**

The model has main effects for two factors (**Container**, with 7 levels for each container tested, and **Treatment**, with two levels: *Single items* and *Multiple containers*), and an interaction term, **Container:Treatment**.

Experiment 1 analysis of deviance

Source Df Deviance Mean deviance F p-Value

Container 6 31521.9 5253.6 57.92 <0.00001 ***

Treatment 1 1446.4 1446.4 15.95 0.00012 ***

Container:Treatment 5 714.5 142.9 1.58 0.174 NS

Residual 101 90.7

Dispersion parameter is residual mean deviance


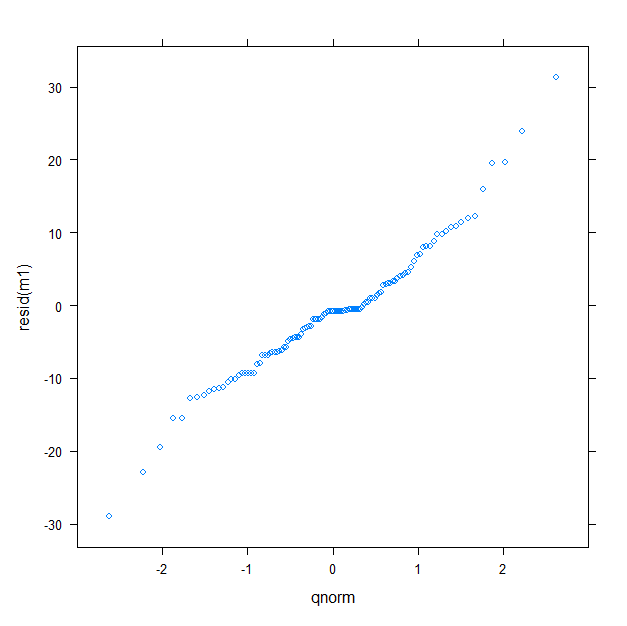


QQ plot of residuals from model, showing a good approximation to a normal distribution

Multiple comparison table

| Linear Hypothesis | Log-scale | pvalue |
| --- | --- | --- |
| singleitembottle - singleitemtyre | -3.88435573 | <0.0001 |
| singleitembottle - singleitemplant | -4.25003836 | <0.0001 |
| singleitembottle - mulitpleitemtyre | -3.22184245 | <0.0001 |
| singleitembottle - mulitpleitemplant | -3.89380423 | <0.0001 |
| singleitemdrain - singleitembucket | -3.57677593 | <0.0001 |
| singleitemdrain - singleitemtyre | -5.89218009 | <0.0001 |
| singleitemdrain - singleitemplant | -6.25786271 | <0.0001 |
| singleitemdrain - mulitpleitembucket | -3.25581081 | <0.0001 |
| singleitemdrain - mulitpleitemtyre | -5.22966681 | <0.0001 |
| singleitemdrain - mulitpleitemplant | -5.90162858 | <0.0001 |
| singleitemwatertank - singleitemtyre | -4.45205727 | <0.0001 |
| singleitemwatertank - singleitemplant | -4.8177399 | <0.0001 |
| singleitemwatertank - mulitpleitemtyre | -3.78954399 | <0.0001 |
| singleitemwatertank - mulitpleitemplant | -4.46150576 | <0.0001 |
| singleitembucket - mulitpleitembottle | 3.62182761 | <0.0001 |
| singleitemtyre - mulitpleitembottle | 5.93723177 | <0.0001 |
| singleitemtyre - mulitpleitemdrain | 5.44895964 | <0.0001 |
| singleitemtyre - mulitpleitemwatertank | 3.54130216 | <0.0001 |
| singleitemplant - mulitpleitembottle | 6.30291439 | <0.0001 |
| singleitemplant - mulitpleitemdrain | 5.81464226 | <0.0001 |
| singleitemplant - mulitpleitemwatertank | 3.90698479 | <0.0001 |
| singleitemplant - mulitpleitemsaucer | 3.40318377 | <0.0001 |
| mulitpleitembottle - mulitpleitembucket | -3.30086249 | <0.0001 |
| mulitpleitembottle - mulitpleitemtyre | -5.27471849 | <0.0001 |
| mulitpleitembottle - mulitpleitemplant | -5.94668026 | <0.0001 |
| mulitpleitemdrain - mulitpleitemtyre | -4.78644636 | <0.0001 |
| mulitpleitemdrain - mulitpleitemplant | -5.45840813 | <0.0001 |
| mulitpleitemwatertank - mulitpleitemplant | -3.55075065 | <0.0001 |
| singleitembucket - mulitpleitemdrain | 3.13355548 | 0.0001 |
| singleitemtyre - mulitpleitemsaucer | 3.03750115 | 0.0001 |
| singleitemplant - mulitpleitembucket | 3.0020519 | 0.0001 |
| mulitpleitemsaucer - mulitpleitemplant | -3.04694964 | 0.0001 |
| singleitemdrain - mulitpleitemsaucer | -2.85467894 | 0.0003 |
| mulitpleitembottle - mulitpleitemsaucer | -2.89973062 | 0.0003 |
| mulitpleitemwatertank - mulitpleitemtyre | -2.87878888 | 0.0003 |
| mulitpleitemdrain - mulitpleitembucket | -2.81259036 | 0.0004 |
| singleitembucket - singleitemplant | -2.68108679 | 0.001 |
| mulitpleitembucket - mulitpleitemplant | -2.64581777 | 0.0012 |
| singleitemtyre - mulitpleitembucket | 2.63636928 | 0.0013 |
| mulitpleitemdrain - mulitpleitemsaucer | -2.41145849 | 0.0049 |
| mulitpleitembottle - mulitpleitemwatertank | -2.3959296 | 0.0053 |
| mulitpleitemsaucer - mulitpleitemtyre | -2.37498787 | 0.0058 |
| singleitemdrain - mulitpleitemwatertank | -2.35087792 | 0.0065 |
| singleitembucket - mulitpleitemplant | -2.32485265 | 0.0074 |
| singleitembucket - singleitemtyre | -2.31540416 | 0.0076 |
| singleitembottle - mulitpleitembottle | 2.05287603 | 0.0325 |
| singleitembottle - singleitemdrain | 2.00782435 | 0.0401 |
| mulitpleitembucket - mulitpleitemtyre | -1.973856 | 0.0465 |
| singleitemwatertank - singleitembucket | -2.13665311 | 0.0625 |
| mulitpleitemdrain - mulitpleitemwatertank | -1.90765748 | 0.0625 |
| singleitembucket - mulitpleitemtyre | -1.65289088 | 0.2075 |
| singleitemwatertank - mulitpleitembucket | -1.81568799 | 0.2288 |
| singleitembottle - singleitembucket | -1.56895157 | 0.2831 |
| singleitembottle - mulitpleitemdrain | 1.5646039 | 0.2831 |
| singleitemwatertank - mulitpleitembottle | 1.4851745 | 0.7284 |
| singleitemdrain - singleitemwatertank | -1.44012282 | 0.8192 |
| singleitemwatertank - mulitpleitemsaucer | -1.41455612 | 0.8568 |
| singleitembottle - mulitpleitembucket | -1.24798646 | 0.8742 |
| singleitembucket - mulitpleitemwatertank | 1.225898 | 0.906 |
| singleitembottle - singleitemwatertank | 0.56770154 | 1 |
| singleitembottle - mulitpleitemwatertank | -0.34305357 | 1 |
| singleitembottle - mulitpleitemsaucer | -0.84685459 | 1 |
| singleitemdrain - mulitpleitembottle | 0.04505168 | 1 |
| singleitemdrain - mulitpleitemdrain | -0.44322045 | 1 |
| singleitemwatertank - mulitpleitemdrain | 0.99690237 | 1 |
| singleitemwatertank - mulitpleitemwatertank | -0.91075511 | 1 |
| singleitembucket - mulitpleitemsaucer | 0.72209699 | 1 |
| singleitembucket - mulitpleitembucket | 0.32096512 | 1 |
| singleitemtyre - singleitemplant | -0.36568262 | 1 |
| singleitemtyre - mulitpleitemtyre | 0.66251328 | 1 |
| singleitemtyre - mulitpleitemplant | -0.00944849 | 1 |
| singleitemplant - mulitpleitemtyre | 1.02819591 | 1 |
| singleitemplant - mulitpleitemplant | 0.35623413 | 1 |
| mulitpleitembottle - mulitpleitemdrain | -0.48827213 | 1 |
| mulitpleitemwatertank - mulitpleitemsaucer | -0.50380102 | 1 |
| mulitpleitemwatertank - mulitpleitembucket | -0.90493289 | 1 |
| mulitpleitemsaucer - mulitpleitembucket | -0.40113187 | 1 |
| mulitpleitemtyre - mulitpleitemplant | -0.67196177 | 1 |

**Experiment 2 – 1-way analysis of deviance, quasi-poisson GLM**

The statistical model has a single factor, **Container,** with 5 levels, one for each container used.

Source Df Deviance Meandeviance F p-Value

Container 4 3838.3 959.6 11.65 0.00002 ***

Residual 25 82.3

Dispersion parameter is residual mean deviance

QQ plot of model residuals shows a good approximation to a normal distribution.
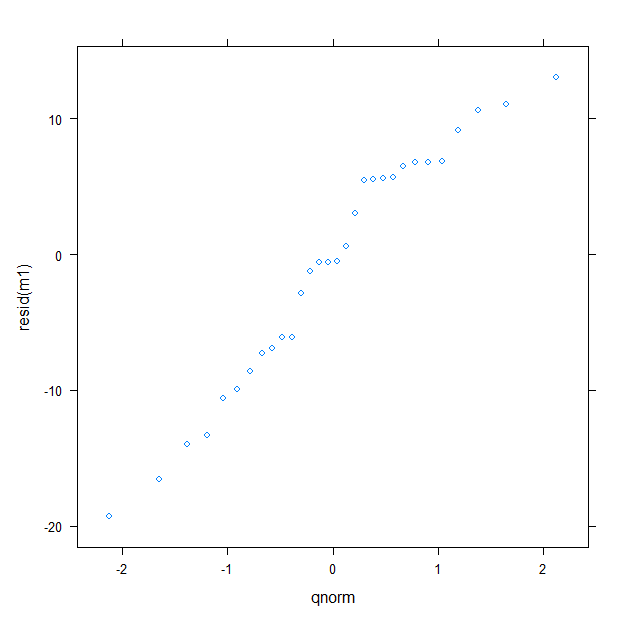

Supplement: Supplementary file 1 — Data S1. Supporting Information [file MVE-37-683-s002.docx]
