# Supplementary material for: Aedes aegypti oviposition‐sites choice under semi‐field conditions
Source: Med Vet Entomol. 2023 Jun 2;37(4):683–92. doi: 10.1111/mve.12670 (PMC10946600; doi:10.1111/mve.12670)
Supplement: Supplementary file 2 — Data S2. Supporting Information [file MVE-37-683-s001.pdf]

STRUCTURED REFLEXIVITY STATEMENT FOR INTERNATIONAL RESEARCH PARTNERSHIPS

Please complete all sections relevant to your research study

Study conceptualisation	
Please justify the choice to conduct the study in and/or acquire samples from the LMIC location	*The study was conducted in Brazil were Aedes aegypti can be found.*
How does this study address local research priorities and how were local researchers involved in study design?	*Brazil is endemic for arboviruses (dengue, Zika and chikungunya) transmitted by Ae. aegypti mosquitoes.*
Research management	
How has funding been used to support the local research team(s)?	*To acquire materials and reagents for experiments and support scientific meetings.*
Data acquisition and analysis	
How are research staff who conducted data collection acknowledged?	*Research staff who collect the data are among the study authors (MRD, RMF and MTP).*
How have members of the research partnership been provided with access to study data and analytical tools?	*They had full access to data and tools via folders shared online.*
If genetic resources were shared, how were the principles of the Nagoya Protocol on the equitable sharing of benefits	*No genetic resources were shared.*
Data interpretation	
How have research partners collaborated in interpreting study data?	*SY was responsible for statistical analysis, MRD and GG prepared the figures and tables and all authors discussed the results.*
Drafting and revising for intellectual content	
How were research partners supported to develop writing skills?	*Authors from LMIC have sufficient writing skills to write a scientific paper.*
Authorship	
How is the leadership, contribution and ownership of this work by LMIC researchers recognised within the authorship?	*MRD led the data collection and writing of the article, so she is the corresponding author. RMF and MTP participated in data collection, analysis and discussion of results and are also authors of the study.*
How have early career researchers across the partnership been included within the authorship team?	*MRD (corresponding author) and MTP are early career researchers from Brazil.*
How has gender balance been addressed within the authorship?	*From the nine authors, four are woman.*
Training	
How has the project contributed to training of LMIC researchers?	*The Brazilian team is composed of researchers that are world leading specialists in the ecology of disease vectors, particularly Aedes mosquitoes, and thus received no training from the HIC researchers.*

Adapted from: Morton et al. 2021 "Consensus statement on measures to promote equitable authorship in the publication of research from international partnerships" https://doi.org/10.1111/anae.15597
